# Study of the Effect of Nutrient Environment on the Biological Characteristics of *Fusarium solani* Clinical Corneal Isolates

**DOI:** 10.1155/2021/6666303

**Published:** 2021-09-23

**Authors:** Dalan Jing, Yingyu Li, Ziyuan Liu, Chen Huang, Pei Zhang, Wei Wang

**Affiliations:** ^1^Department of Ophthalmology, Peking University Third Hospital, Beijing, China; ^2^Beijing Key Laboratory of Restoration of Damaged Ocular Nerve, Peking University Third Hospital, Beijing, China

## Abstract

**Purpose:**

The biological characteristics of *Fusarium solani* clinical corneal isolates growing in different nutritional conditions in vitro were studied in order to find out the key point of pathogenicity.

**Methods:**

Five kinds of media with different glucose and nitrogen concentrations were prepared as the liquid and solid forms. The clinical isolates were as follows: 2 *Fusarium solani* strains. The clinical corneal isolates and the standard strains were inoculated in the solid and liquid media. They were all incubated at 296 for 96 h and observed at defined time points. The optical density was recorded to generate the growth curves in liquid media. Morphologic changes of colonies in the solid media were determined under the light microscope.

**Results:**

The clinical isolates of *Fusarium solani* showed stronger reproductive capacity in the abominable nutritional condition. Besides, when the glucose concentration in the medium was consistent with the glucose concentration of aqueous in diabetic patients, the clinical isolates would show the biological features of quicker growth rate and stronger reproductive capacity.

**Conclusions:**

Nitrogen source is essential for fungus reproduction. The clinical isolates showed stronger environmental adaptability under different nutritional conditions and more sensitive to environmental changes.

## 1. Introduction

Ocular fungal infections have been recognized as important causes of blindness and morbidity. It was formerly believed that fungal eye infections were quite rare. However, with the widespread use of antibiotics, glucocorticoid and immunosuppressant, fungal eye infections have become fairly common [1–3]. Keratitis is by far the most frequent fungal infection of the eye [4]. The cornea is relatively in an immune privilege position, and this is due to a series of molecular and cellular pathways [5]. But patients with fungal keratitis are immunocompetent compared with other patients who have pulmonary or systemic fungal infection. Thus, keratitis patients mount a variety of immune reactions to fungi [6, 7]. The clinical outcomes of fungal keratitis are worse than bacterial keratitis due to delay diagnosis, abuse of antibiotics or steroids, fungal virulence, and few antifungal drugs [8]. Fungal keratitis can easily lead to serious consequences; the majority of patients with moderate or worse visual impairment and about 25% patients need corneal transplantation [9]. Fungal keratitis is a major blinding eye disease in Asia [10]. Meanwhile, the morphologic characteristics, growth features, and physiochemical properties of fungi changed substantially after these experiences involve immune attack and intracorneal growth. Clinical corneal isolates might differ from standard strains in many ways.

Previous studies have not demonstrated these changes of clinical corneal isolates. At the same time, the biological characteristics of clinical corneal isolates growing in different nutrition conditions in vitro may be different from standard strains. Further investigations are needed to better understand and treat fungal keratitis.

Fungi belonging to nearly 56 genera have been reported from the cases of corneal infections. The main pathogen causing fungal keratitis have been shown to be geographically distributed. In low-income or middle-income countries, the most common causative organisms of fungal keratitis are *Fusarium* [11]. In high-income countries, *Candida* was much more common [2]. With much of this development increasing the popularity of contact lenses, mycotic keratitis associated with *Fusarium* may also be on the rise. Thus, reinforcing the study of *Fusarium* is a top priority, and the clinical corneal isolates of *Fusarium* are more important. In clinical practice, most of the isolates of *Fusarium* keratitis were *Fusarium solani* (*F. solani*). [12]

In our study, we chose to study *F. solani* isolated from corneas and standard strain of *F. solani.* We explored the differences between these in biological characteristics by changing nutritional conditions in vitro and to discover the key point of pathogenicity and provide a new idea for the treatment of fungal keratitis.

## 2. Materials and Methods

### 2.1. Fungal Strains and Cultivation Methods

We have chosen three different *F. solani* strains, including 1 standard strain and 2 clinical corneal isolates. The standard laboratory reference wild-type *F. solani* strain CGMCC 3.5840 was obtained from the China General Microbiological Culture Collection Center (CGMCC). Two clinical corneal isolates, S1 and S2, were obtained from two keratitis patients, stored at the Microbiology Laboratory of Ophthalmology Department, Peking University Third Hospital, China. All the strains were frozen at −20°C. Liquid media were prepared as described in Table 1. Double-strength media were prepared and sterilized by autoclavation (115°C, 20 min) during the experiments. Solid media used double-fold concentration liquid media as described above were diluted 1 : 2. Then, the media were rotated with 2% agarose gels. After autoclavation (115°C, 20 min), media were then poured into peri dishes with a diameter of 90 mm to a thickness of approximately 4 mm. The strains were revived through being cultured on Sabouraud glucose agar (SGA) (bioMerieux, China) media at 29°C for additional 3 days. Conidia and spores were collected using a cotton swab and suspended in 0.1% Tween 80 (Amresco, USA). The suspensions were adjusted to a concentration of 2 × 10^6^ spores/ml by using a hemocytometer cell counting chamber.

### 2.2. Morphology Examination

**Figure 1:**
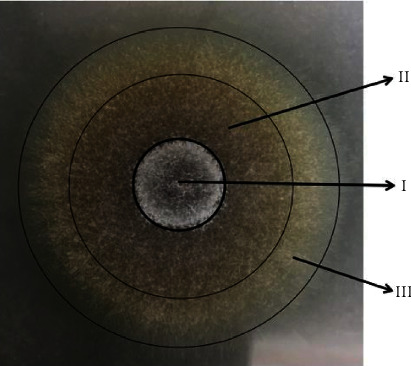
Colonial sections of S2.

The suspension was diluted to 1 × 10^6^ spores/ml density. Each spore suspension of 40 *μ*l was grown on the center of the solid medium. The dishes were incubated at 29°C for 96 h. In gross observation, we recorded the diameter, density, growth velocity, and pigmentation of the colonies at the time points of 0, 12, 24, 36, 48, 60, 72, and 96 h. Morphological and anatomical features of the cultured strains were analyzed under light microscopy and documented by digital photographs. In addition, our previous results show that the colonies of S2 presented an obvious color partition which contained three sections such as I, II, and III part in Sabouraud's medium (Figure 1). Furthermore, the diameter of I, II, III part should be recorded. In order to study the morphology of fungi, mycelial threads and spores were observed microscopically which were harvested from different locations of media: the center, pericenter, and periphery of CGMCC3.5840 and S1, the I, II, and III part of S2 at the time point of 48 and 96 h in the slides. Before microscopic observation, specimens were counterstained with Giemsa stain.

### 2.3. Growth Curves

For this experiment, we used S1 and S2 that were grown on the five different liquid media as the experimental group, CGMCC3.5840 grown on the similar liquid media as the control experimental group, and the uninjected similar liquid media as the empty control group. Fifty microliters of each spore suspension was injected into 50 *μ*l of double-strength liquid medium in 96-well plates. Fifty microliters of 0.1% Tween 80 was added into five empty groups. In all groups, 6 wells were inoculated. The plates were coincubated at 29°C for 96 h. We selected 6 different time points, 0, 12, 24, 36, 48, 60, 72, and 96 h, in order to measure the optical density (OD). OD was read by a spectrophotometer at 405 nm without shaking. Growth curves were generated based on OD changes at different time points. For the purposes of comparing the growth curves for each species in five different media, a variety of parameters were calculated and recorded: the highest OD (OD_max_), the OD change (△OD) (△OD = OD_*t*2_ − OD_*t*1_, △OD_max_ = OD_max_ − OD_intial_), the global slope(S), the maximal slope(*S*_max_), the time at that *S*_max_ of the growth curve was reached (*T*_max_), and the time at which curve slope declined first.

### 2.4. Statistical Analyses

All statistical analyses were performed using SPSS Statistics (IBM, Version 20.0) software. Statistical significance (*p* value <0.05) was assessed by repeated measures ANOVA.

## 3. Results

### 3.1. Correlation of Fungal Colonial Gross Morphology with Type of Growth Media

**Figure 2:**
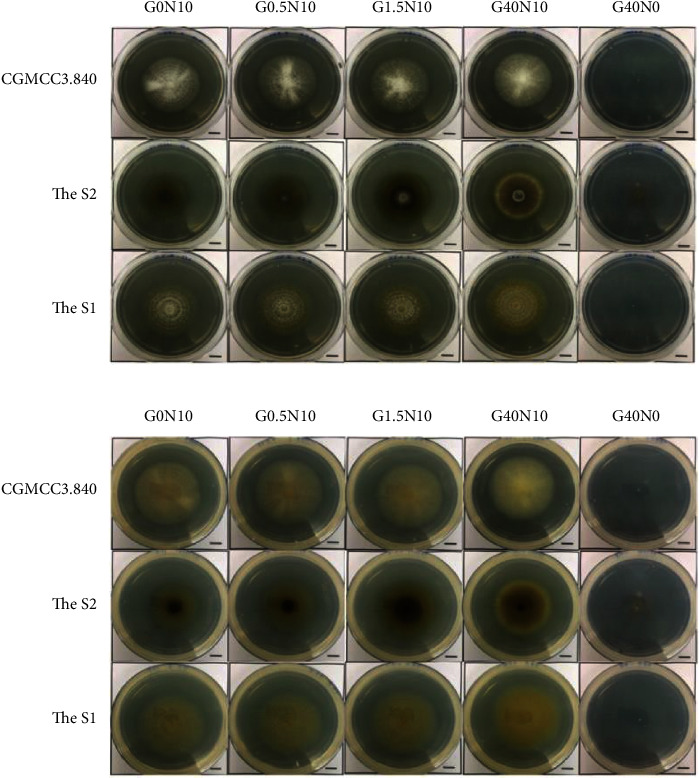
Fungal colonial gross morphology on different solid media at 96 h.

**Figure 3:**
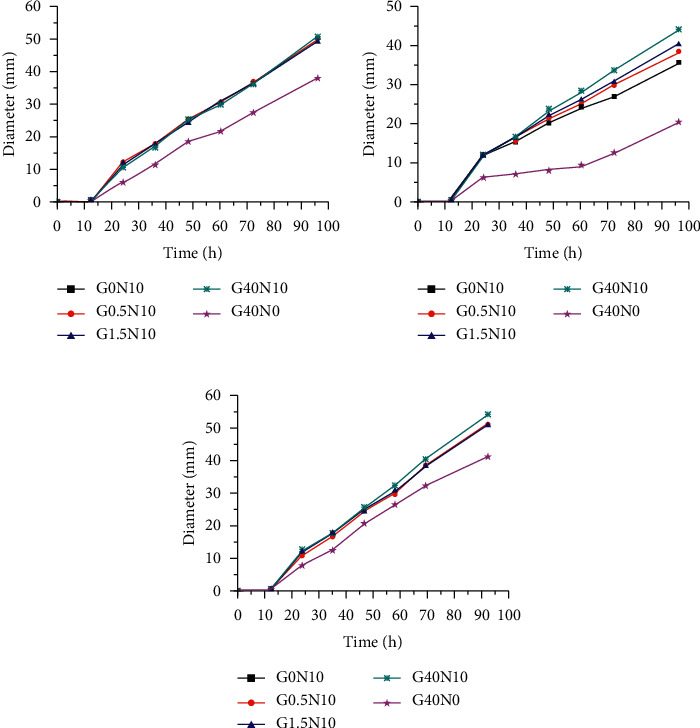
The colonial diameter changing of each strain on different solid media. (a) CGMCC3.5840. (b) S2. (c) S1.

The colonial diameter of each strain was time-varied depending on the different media (Figure 2). We observed that the diameter and density of S1 were more homogeneous with CGMCC3.5840 in solid media. Each of these colonial diameters showed no obvious differences in G0N10-G40N10 media with varying concentration of glucose (Figures 3(a) and 3(c)). The colonial densities of low-glucose media (G0N10, G0.5N10, and G1.5N10) were more sparse than high-glucose media. In low-nitrogen media, the colonial ranges were more lagging than others, and a few hyphae attached to the media was observed. The mean difference between these colonies was color: S1 was light yellow, while CGMCC3.5840 was white.

For S2, clear difference in growth was observed compared to CGMCC3.5840. The culture grossly showed smaller than the standard strains. As the glucose continued to increase, so did the range of colony. In the low-nitrogen media, the colony ranges were smaller than others. Unlike the standard strains, their hyphae grew not only on the surface of the media but also towards the air. Thus, S2 formed significant colonies that presented a color division. In Table 2, we recorded the characteristics of different sections at 96 h.

### 3.2. Correlation of Fungal Colonial Microscopic Morphology with Type of Growth Media

**Figure 4:**
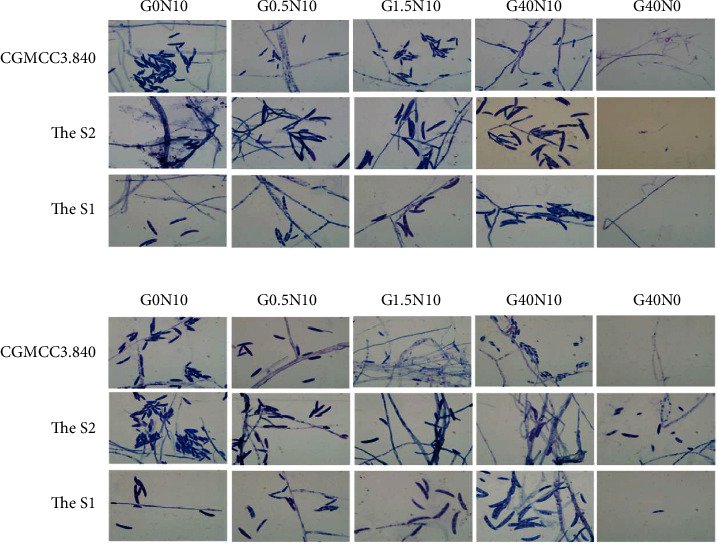
Fungal colonial microscopic morphology on different liquid media (the center of the colony, Giemsa staining, 1000×).

Hour 48 fungal colonies, the standard strain tended to produce larger spores in low-glucose media, while S1 and S2 tended to produce equal-sized relative spores in different glucose concentration media. Spores were observed in the low-nitrogen media of S2; however, only hyphae were observed in S1 and standard strains (Figure 4(a)).

96 hours after culturing, all the strains produced equal-sized relative spores in different glucose concentration media. In the low-nitrogen media, S2 made more spores, S1 produced small amounts of spores, while no spores were visible for the standard strains (Figure 4(b)).

**Figure 5:**
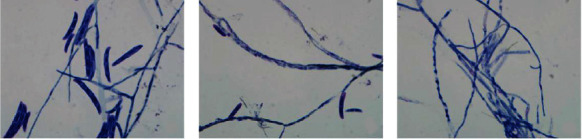
The smear of S2 in different sections (Giemsa staining, 1000×). (a) I part. (b) II part. (c) III part.

In the smears of different sections, the difference of spore counts between the center and pericenter for the standard strains and S1 had no significance. While, hyphae and minor spores were observed in the periphery. For S2, the nearer the center, the larger the spores, and more spores can be observed. Only hyphae could be observed in the periphery (Figure 5).

### 3.3. Effect of Glucose Concentration on Growth Curves

**Figure 6:**
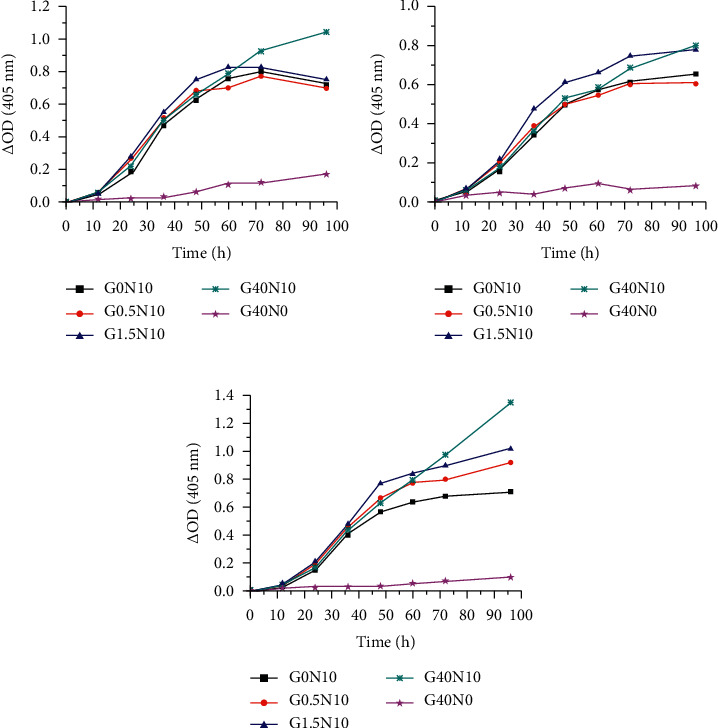
The growth curves of *Fusarium solani* in different liquid media. (a) CGMCC3.5840. (b) S2. (c) S1.

The growth curves were fragmented and analyzed. The strains of CGMCC3.5840 showed major differences in growth curves of differing glucose concentrations (*p* < 0.01) (Figure 6(a)). The standard strains showed no significant change in OD after 0–12 h of incubation in the each medium. The first significant change in OD was after 24–36 h, reaching *S*_max_. The OD of G1.5N10 was larger than the others before 60 h. While, the OD of G40N10 was largest beyond G1.5N10 after 60 h.

For the clinical isolate S2 (Figure 6(b)), there had been significant differences between paired comparisons of growth curves (*p* < 0.01) with the exception of the groups G0N10 and G0.5N10 (*p*=0.64). The S2 showed no significant change in OD after 0–12 h of incubation in each medium. When the time increased to 24–36 h, *S*_max_ was achieved. Meanwhile, S of G1.5N10 was larger than the others. The OD of G1.5N10 was larger than the others before 72 h and increased more slowly. The OD of G1.5N10 appeared to be exceeded near the time point of 96 h.

Unlike S2, S1 showed major differences in growth curves of differing glucose concentration (*p* < 0.01) (Figure 6(c)). They showed no significant change in OD after 0–12 h. When the time increased to 24–36 h, *S*_max_ was achieved. The OD of G1.5N10 was larger than others before 60 h. Next, it was exceeded by G40N10 after 60 h.

**Figure 7:**
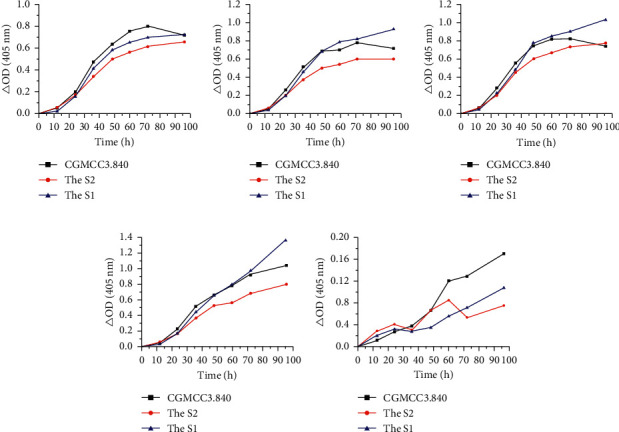
The comparison of growth curves between different strains in the same liquid media. (a) G0N10. (b) G0.5N10. (c) G1.5N10. (d) G40N10. (e) G40N0.

There was a significant difference between the standard strains and clinical isolates regarding growth curves in the same media (*p* < 0.01) (Figure 7). The ODs of the standard strains were larger than the clinical isolates in G40N0. Except that, the OD of S2 was relatively lower than other strains in other media.

### 3.4. Effect of Nitrogen on Growth Curves

For the standard strains or clinical isolates, the ODs of the low-nitrogen media were significantly lower than others (*p* < 0.01). As the time prolonged, the OD increased more slowly.

## 4. Discussion

Studies have documented that the growth curve can be well used to describe each stage of fungal development [13]- △OD was more accurate than gross inspection that could directly dictate the development of strains. While the morphologic observation had advantages of intuition and specificity. We could reveal the growth properties of strains more comprehensive through a combination of two methods. Progressively, the similarities and differences were compared between the standard strains and clinical corneal isolates.

The nutritional metabolic capacity of fungus, especially the utilization of glucose and nitrogen, plays an essential role in their ability to cause disease and invasive ability [14]. The carbon source and the nitrogen source, which serve as the major energy for fungal growth, most need to be obtained from the external environment [15]. We suggest that changes in nutritional environment, particularly in carbon and nitrogen source, may lead to the changes of fungal growth properties.

Due to clinical corneal isolates, the main growth environment is the cornea whose glucose suppliers are the blood vessels of limbus and aqueous humor. Diabetes mellitus increases their blood glucose concentration and aqueous humor glucose concentration compared with nondiabetic patients [16]. In our experiment, the formulation of G40N10 was made according to Sabouraud's medium. The glucose concentration of G0.5N10 referred to aqueous humor glucose concentration of the normal person, while G1.5N10 referred to the diabetes mellitus [16]. To explore the impact of glucose on the growth of clinical corneal isolates, we observed the growth state of the standard strains and clinical corneal isolates in different glucose concentration media through a series of graded concentrations of glucose. One of the main constituents of the cornea is collagen that is rich in protein (nitrogen). For the purpose of identify the effects of nitrogen on clinical corneal isolates growth, we set G40N0 nitrogen-free media to compare with G40N10.

### 4.1. Effect of Nitrogen on Clinical Corneal Isolates

Nitrogen plays an important role in clinical corneal isolates growth. Each strain maintained a comparatively well growing condition in the absence of glucose. In contrast, neither the standard strains nor the clinical corneal isolates remained in slow growing condition in nitrogen-free media. We suggested protein can serve as a carbon source in fungal growth [14]. However, the glucose may not provide nitrogen source to fungal growth.

Although each strain was slow growing in G40N0, the clinical corneal isolates were superior to the standard strains for growth capacity in the solid media. The spores could not be seen in the group of standard strains at any time point. For the clinical isolate S2, although their colonial diameters were less than the standard strains, their colonial densities were larger. Moreover, their hyphae grew towards the air and appeared light yellow-brown on the solid media. The spores could be seen under the microscope at the time point of 48 h. The S1 and standard strains were almost identical in growth condition by macroscopic observation of G40N0. At 96 h, the spores should be visible under light microscopy.

The nitrogen was necessary for fungal growth. Previous studies have shown that when the *Aspergillus fumigatus* cannot obtain all the nutrients, its survival depends upon the cross-pathway control (CPC) system which is stimulated by lack of amino acids [17]. Literature review does not provide strong clues for a similar CPC system in *Fusarium solani.* More studies are needed to find the effective route of nitrogen source using for *Fusarium solani.*

### 4.2. Effect of Glucose on Clinical Corneal Isolates

When the supply of nitrogen was constant, effects of glucose concentration changes on each strain were shown. Different sources and different experiences of strains may have different responses to glucose concentration changes.

Early in culture, the *Fusarium solani* had the most rapidly rising growth in liquid media of G1.5N10 for the standard strains and the clinical corneal isolates. While the growth of G1.5N10 entered a plateau phase or a slowly growing phase, the OD of G40N0 was larger than G1.5N10. We suggest that total quantity of nutrients was limited, and the optimum growing condition is aqueous glucose concentration of DM. Our previous findings suggest that recurrent fungal infections of DM in 2 cases were affected by *Fusarium solani.* The growth curves analysis showed that all three curves of the CGMCC3.5840 had no significant difference in the media whose glucose concentrations were 0–1.5%, the growth curve of S2 in G1.5N1.0 was significantly higher the other curves, and the three curves of S1 had a significant gradient difference. All of these results have shown that the clinical corneal isolates' perception of glucose concentration was significantly superior to the CGMCC3.5840.

The perception of S2 was displayed evidently in solid media with different glucose concentrations. Additionally, as the glucose concentrations increased, the colonial diameters became enlarged and the colonial densities increased. Compared to S1 and CGMCC3.5840, the backside of the S2 culture was the deepest. The combinatorial analysis indicated that the more complex environmental experience the strains had, the deeper color the culture had. The fungal pigmentation, especially the melanins are associated with virulence and increase the fungal abilities of aggressiveness and resistance. More evidence indicates that active melanin formation appears to protect *Penicillium marneffei*, *Aspergillus fumigatus*, and *Cryptococcus neoformans* by enhancing these resistance and aggressiveness [18, 19]. As reported by Chiewchanvit et al., *Fusarium keratoplasticum* (a member of *F. solani* species complex) is isolated from a patient with systemic fusariosis; its hyphae and chlamydospores can produce melanin in vitro and that hyphae can synthesize melanin in vivo. All of these characteristics may play an important role in pathogenicity. [20]

We hypothesized that the fungal pigmentation might also play an essential rule in pathogenicity for the clinical corneal isolates, and the deeper the color, the higher pathogenicity the fungus should have, leading to recurrent infection of S2. Conversely, complex environment caused S2 to generate more pigmentation for defencing environmental changes. In our research, the backside of the culture was larger in G1.5N10 than others. It was hypothesized that S2 has the strongest invasiveness in nutritional environment of DM. Further analyses with background and derivation of strains were done: the CGMCC3.5840 with a complex environmental experience, the S1 was obtained from the patient of traumatic fungal corneal ulcer, and the S2 was obtained from the recurrent fungal corneal ulcer one with DM which might be correlated to recurrence and experienced 2 months antifungal agents treatment. Thus, long-term exposure to antifungal agents made the growth state of S2 lower than others in the no-nitrogen media. But, S2 appeared to be more adaptable in the harsh environment (G40N0). Because of deriving from DM, S2 was more sensitive to glucose concentrations and showed stronger growth capacity and invasiveness. Clinical experience of the clinical corneal isolates might confer a fitness advantage. The clinical isolates showed stronger environmental adaptability and faster perception of environmental change. Accordingly, we hypothesized that by adjusting microenvironment and supplementing with specific antifungal agents, better results may be obtained. It is a new treatment for corneal fungal infection.

Nitrogen source is a very important nutrient element for fungus growth. The clinical isolates have shown stronger growth capacity and reproduction on no-nitrogen media. The clinical isolates have stronger environmental adaptability and could faster perceive the glucose changes in rich nitrogen condition. The cause of these differences needs further investigation. In addition to the human immune attacks, the clinical isolates underwent complex environment, such as antifungal agents. All the factors might cause genomic mutations or gene expression changing. Genome studies or functional protein studies need to be used to test it.

At present, there are no specific drugs for fungus of different genera, and the animal model of pharmacology was infected by the standard strains. Our study demonstrated that clinically, fungus infection of different genera had different biological characteristics in vivo. This was observed in vitro as well. Besides, the standard strains and the clinical isolates of same genus were also different in biological features. Emerging evidence suggested that the differences between genera should be noted in the pharmacologic study, and more clinical isolates should be used in research. Only in this way can more specific antifungal agents be generated.

## 5. Conclusion

Nitrogen source plays an important role in fungal growth. On the one hand, it may be possible to treat fungal infections by changing the content of nitrogen sources in the corneal microenvironment. One the other hand, we might prevent fungal growth by inhibiting gene or enzyme that utilize the nitrogen source. It provides a new breakthrough in the treatment of fungal keratitis. The clinical isolates of *Fusarium solani* were more sensitive to environmental changes-glucose concentration and shown stronger growth capacity and invasiveness. Clinical experience of the clinical corneal isolates might confer a fitness advantage. More studies about the clinical corneal isolates are needed to treat fungal infection.

## Figures and Tables

**Table 1:** Formulation of different liquid media.

Culture medium	Carbon source, glucose (g)	Nitrogen source, peptone (g)	Distilled water (ml)
G0N10	0	10	1000
G0.5N10	0.5	10	1000
G1.5N10	1.5	10	1000
G40N10	40	10	1000
G40N0	40	0	1000

**Table 2:** The colonial features of S2 in each solid medium.

Culture medium	Diameter (mm)	Diameter of I part (mm)	Width of II part (mm)	Width of III part (mm)	The back of I part	The back of II part	The back of III part
G0N10	37	No significant division between I and II, totally 12		25	Dark brown	Dark brown	Light brown
G0.5N10	40	No significant division between I and II, totally 12		28	Dark brown	Dark brown	Light brown
G1.5N10	42	8	14	20	Dark brown	Dark brown	Light brown
G40N10	46	11	22	13	Dark brown	Light brown	Yellow-white
G40N0	21	No significant division					

## Data Availability

The data used to support the findings of this study are available from the first author upon request.
